# Diverse roles of Dpb2, the non-catalytic subunit of DNA polymerase ε

**DOI:** 10.1007/s00294-017-0706-7

**Published:** 2017-05-17

**Authors:** Michał Dmowski, Iwona J. Fijałkowska

**Affiliations:** Institute of Biochemistry and Biophysics, Polish Academy of Sciences, Pawińskiego 5a, 02-106 Warsaw, Poland

**Keywords:** Dpb2, Polymerase ε, Cell cycle, MBF transcription factor

## Abstract

Timely progression of living cells through the cell cycle is precisely regulated. This involves a series of phosphorylation events which are regulated by various cyclins, activated in coordination with the cell cycle progression. Phosphorylated proteins govern cell growth, division as well as duplication of the genetic material and transcriptional activation of genes involved in these processes. A subset of these tightly regulated genes, which depend on the MBF transcription factor and are mainly involved in DNA replication and cell division, is transiently activated at the transition from G1 to S phase. A *Saccharomyces cerevisiae* mutant in the Dpb2 non-catalytic subunit of DNA polymerase ε (Polε) demonstrates abnormalities in transcription of MBF-dependent genes even in normal growth conditions. It is, therefore, tempting to speculate that Dpb2 which, as described previously, participates in the early stages of DNA replication initiation, has an impact on the regulation of replication-related genes expression with possible implications for genomic stability.

DNA replication is a tightly controlled process which occurs only once per cell cycle. Moreover, the duplication of the genetic material has to be coordinated with the cell growth and division. Thus, multiple mechanisms have evolved to ensure proper timing of all processes throughout all cell cycle phases. This includes the control of replisomes assembly at the origins of replication and replication arrest in response to perturbations in DNA synthesis (Hustedt et al. 2013; Gadaleta et al. 2016). In eukaryotic cells, high fidelity duplication of the genetic material is performed by three main multisubunit DNA polymerases: the polymerase α (Polα) synthesizes primers which are extended by Polε or Polδ, the two major replicative polymerases. Polε is postulated to be the leading strand polymerase, for review see (Lujan et al. 2016) and here we will focus on this polymerase.

Polε is composed of the catalytic subunit Pol2 and three non-catalytic subunits Dpb2, Dpb3 and Dpb4. Although the N-terminal part of Pol2 is dispensable, its C-terminal half is essential for cell survival (Dua et al. 1999; Isoz et al. 2012). This region of Pol2 interacts with the other essential subunit Dpb2, which in turn binds Psf1, a subunit of the GINS complex (Takayama et al. 2003; Sengupta et al. 2013). GINS is a multiprotein complex composed of Psf1, Psf2, Psf3 and Sld5 subunits (Takayama et al. 2003) which, together with Cdc45 and Mcm2-7, form the CMG helicase complex essential in both the initiation and elongation of DNA replication (Moyer et al. 2006). Therefore, Dpb2, which links the Polε with the CMG complex, is important for the assembly of the replisome and targeting of Polε to the leading strand (Langston et al. 2014; Grabowska et al. 2014). Mutations in *DPB2* allels *dpb2*-*100* or *dpb2*-*103* affect the interaction of Dpb2 not only with Pol2 (Jaszczur et al. 2008) but also with Psf1 and Psf3 subunits of the GINS complex (Garbacz et al. 2015; Dmowski et al. 2017). Dpb2 dysfunction in *dpb2*-*100* or *dpb2*-*103* mutants results in DNA replication elongation defects, prolonged S phase and increased spontaneous mutagenesis (Jaszczur et al. 2008). Importantly, mutations in *DPB2* or deletion of this gene do not influence the catalytic properties of Polε (Ganai et al. 2015; Garbacz et al. 2015).

## Polymerase ε and the S phase checkpoint

**Fig. 1:**
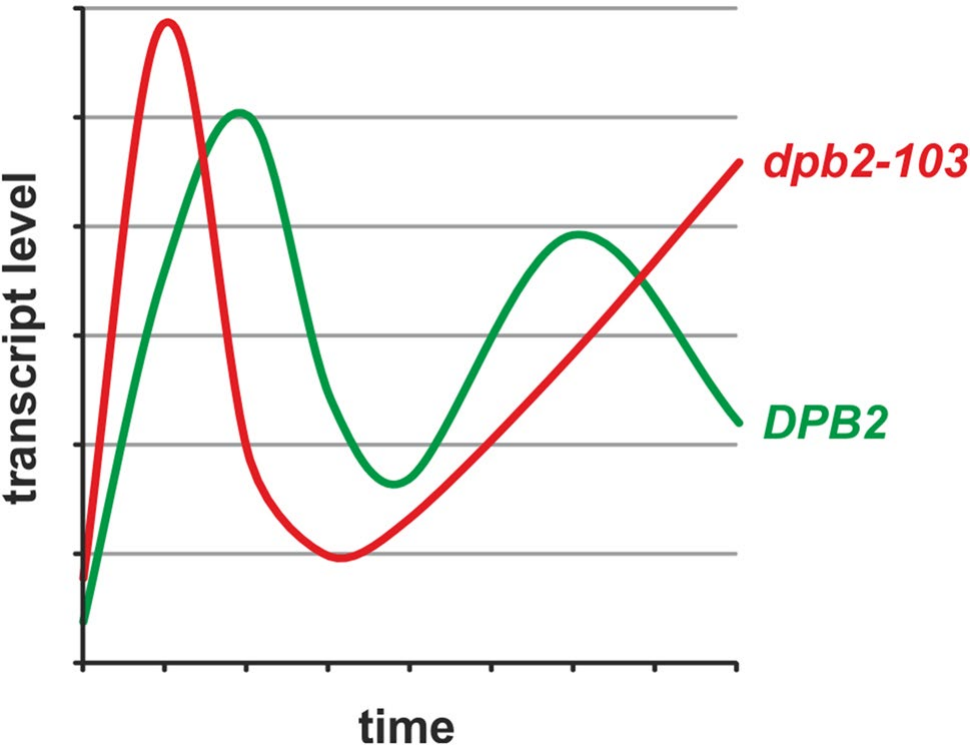
Simplified graph presenting the transcript levels of MBF-regulated G1/S transition genes in a wild type and *dpb2*-*103* cells after the release from G1 block. Based on Fig. 6 from (Dmowski et al. 2017)

The S phase checkpoint is activated when DNA replication is threatened (Hustedt et al. 2013; Skoneczna et al. 2015; Palou et al. 2016) and it has been postulated that besides its role in DNA synthesis, Polε is also involved in this process. Early studies suggested that *pol2*-*11* and *pol2*-*12* mutants in the Pol2 C-terminus are impaired in correct response to the replication stress (Navas et al. 1995; Dua et al. 1998). However, the mechanism has not been identified because C-terminal mutations in the catalytic subunit Pol2 give very sick cells and their characterization is difficult. Recently, the involvement of the essential non-catalytic subunit Dpb2 in the response to the replication stress has been demonstrated by studies of the *dpb2*-*103* mutant (Dmowski et al. 2017). Yeast cells, when challenged with the RNR inhibitor hydroxyurea, activate the Rad53 checkpoint kinase, which in turn activate the Dun1–Crt1 branch (mainly dNTP upregulation) and the MBF-Nrm1 branch, which stimulates the transcription of G1/S transition genes (explained below) (Bastos de Oliveira et al. 2012; Hustedt et al. 2013). The MBF-Nrm1-dependent genes analyzed in the *dpb2-103* mutant are involved in environmental stresses response, sister chromatide cohesion, chromosome condensation or morphogenesis (Smolka et al. 2012). In contrast to the wild type cells, *dpb2*-*103* cells treated with hydroxyurea fail to activate the MBF-Nrm1 branch of the replication checkpoint. Thus, in this mutant, G1/S MBF-dependent genes are repressed in the S phase regardless of the hydroxyurea treatment. Another interesting observation comes from the experiments performed during unperturbed growth. In *dpb2*-*103* cells transcriptional activation of MBF-dependent G1/S transition was faster and, more importantly, prematurely switched off, when compared to the wild type cells (Fig. 1) (Dmowski et al. 2017). Thus these observations may be the starting point for investigations of the effect of mutations in the Dpb2 subunit on the transcription of G1/S transition genes and its consequences for the cell cycle progression and genome stability.

## Cell cycle regulation by cyclin-dependent kinases

Expression of G1/S transition genes, like other cell cycle-related processes in eukaryotes, is tightly regulated by highly conserved cyclin-dependent kinases (CDKs) which are activated by cell cycle-specific cyclins. In *Saccharomyces cerevisiae* the Cdc28/Cdk1 kinase is controlled by nine periodically expressed cyclins: three G1 cyclins (Cln1-3) and six B-type cyclins (Clb1-6). This enables thigh control of cellular processes such as DNA replication, cell growth and division, as well as the transcriptional control of genes involved in these processes (Koch et al. 1996; Tanaka et al. 2007a; Benanti 2016; Deshmukh et al. 2016).

**Fig. 2:**
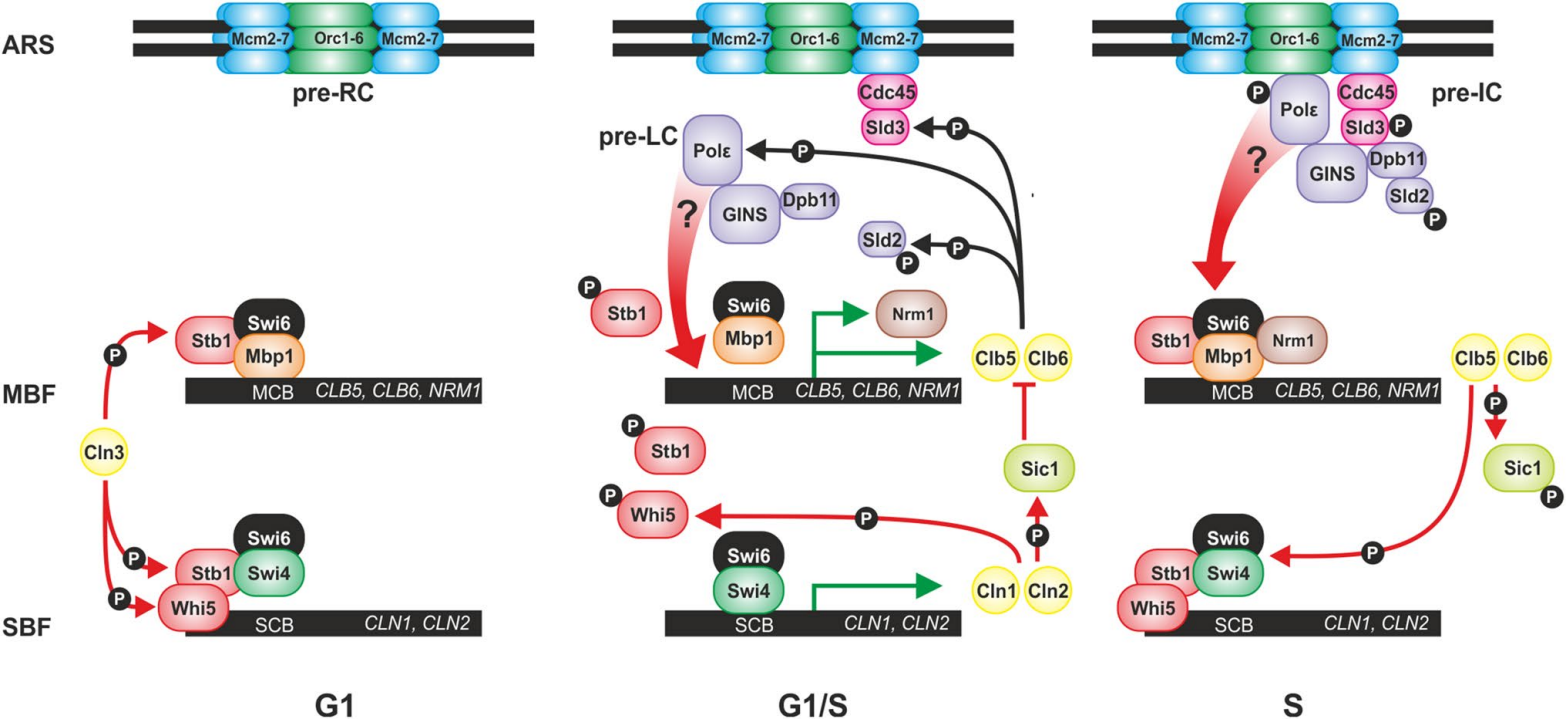
Cell cycle-related events at the origins of replication (ARS) and at promoter regions of G1/S transition genes regulated by MBF and SBF transcription factors.* Thin arrows* denote phosphorylation events which have inhibiting (*red*) and activating (*black*) effects. *Green arrows* denote active transcription. Details are given in the text

In early G1 phase transcription of G1-specific genes involved in transition to the S phase is inactive. These genes are controlled by two transcription factors, the SBF (SCB-binding factor) activator and the MBF (MCB-binding factor) repressor composed of DNA-binding subunits Swi4 and Mbp1, respectively, and a common regulatory subunit Swi6 (Iyer et al. 2001). The genes regulated by the SBF are mainly involved in cell cycle progression (e.g. *CLN1* and *CLN2*) while MBF regulates the genes involved in DNA replication and repair (de Bruin et al. 2006). The MBF repressor with the Nrm1 corepressor interacting with Swi6, remains bound to specific promoter sequences throughout the cell cycle, while the transcription of SBF-dependent genes is inactivated by the transcriptional inhibitor Whi5 bound to SBF from M to late G1 phase (Fig. 2) (Koch et al. 1996; de Bruin et al. 2006). As the cell progress through G1 phase, Cdc28 is activated through binding of the Cln3 cyclin. The Cln3–Cdc28 complex phosphorylates the transcriptional inhibitor Whi5, to promote its dissociation from SBF and thus, the transcriptional activation of dozens of G1-specific genes involved in the transition to the S phase (Bertoli et al. 2013). SBF activation is reinforced by a positive feedback loop where Whi5 phosphorylation by Cdc28 is further activated by two SBF-dependent early-expressed Cln1 and Cln2 cyclines (Eser et al. 2011) whose expression peaks at G1-S transition (Harris et al. 2013). Additionally, both SBF- and MBF-dependent promoters are activated through Cln3–Cdc28-dependent phosphorylation of Stb1 (mediating early G1 repression) which then dissociates from MBF- and SBF-dependent promoters (Fig. 2) (de Bruin et al. 2008; Ferrezuelo et al. 2010). At this stage, cells are committed to pass START and enter the cell cycle (Doncic et al. 2011).

The cell entry into S phase requires Clb5 and Clb6 cyclins whose MBF-regulated expression peaks at G1-S transition. However, the activation of Clb5–Cdc28 and Clb6–Cdc28 complexes requires the ubiquitin-dependent degradation of Sic1, the inhibitor of these complexes. Degradation of Sic1 is promoted not only by Cln1,2–Cdc28 but also by Clb5–Cdc28 through a positive feedback (Nash et al. 2001; Kõivomägi et al. 2011). Clb5 and Clb6 cyclins activate Cdc28 to phosphorylate the Swi4–Swi6 complex which then dissociates from SBF-dependent promoters to inactivate theme (de Bruin et al. 2008). In parallel, MBF-dependent promoters are inactivated by the accumulating Nrm1 repressor (expressed in an MBF-dependent manner), presumably stabilized by Cdc28-dependent phosphorylation (Fig. 2) (de Bruin et al. 2006; Ostapenko and Solomon 2011). However, if the replication stress checkpoint is activated, MBF-dependent transcription is maintained during the S phase (Bastos de Oliveira et al. 2012; Travesa et al. 2012).

In parallel, Clb5,6–Cdc28 also activate the DNA replication. First, the origins of replication (ARS—autonomously replicating sequences) are bound by the ORC (origin recognition complex) composed of Orc1-6 proteins (Liang and Stillman 1997). Next, from late M to G1 phase, the ORC assisted by the Cdc6 ATPase and Cdt1 (the DNA-licensing factor) recruits the inactive Mcm2–7 (minichromosome maintenance) helicase complex to form the pre-RC (pre-replicative complex), reviewed in (Li and Araki 2013). Then, in the late G1 phase, at early-firing origins, DDK (Dbf4-dependent kinase) phosphorylates Mcm2–7, which then recruits Cdc45 together with Sld3 (Kamimura et al. 2001; Araki 2010; Sheu and Stillman 2010). Further steps of DNA replication initiation are regulated by Clb5- and Clb6-dependent Cdc28 (Tanaka et al. 2007a; Tanaka and Araki 2010). First, Clb5,6-dependent phosphorylation of Sld2 by Cdc28 promotes the association of the pre-LC (pre-loading complex) composed of Polε, GINS, Dpb11 and Sld2 (Masumoto et al. 2002). Next, Clb5,6-dependent phosphorylation of Sld3 recruits the pre-LC to the pre-RC to form the pre-IC (pre-initiation complex) (Tanaka et al. 2007b; Zegerman and Diffley 2007). Since Sld2 phosphorylation promotes its association with Dpb11 and Polε but not GINS, it has been suggested that Polε is necessary for the association of GINS with Sld2–Dpb11 (Araki 2010). Therefore, it has been postulated that Pol2–Dpb2 interaction is involved in the process of pre-LC assembly (Muramatsu et al. 2010). Moreover, Dpb2 has been shown to interact with Orc1 and Orc4 from the ORC complex (Krogan et al. 2006).

## Involvement of Dpb2 in regulation of processes at the G1/S transition

In the G1 phase, the Dpb2 subunit of Polε is phosphorylated in a Cln–Cdc28-dependent manner, while in S phase through the activity of Clb5,6–Cdc28 (Kesti et al. 2004). Phosphorylation of Dpb2 is not only independent from Pol2 binding but also enhances its association with Pol2. Therefore, it has been suggested, that phosphorylation of Dpb2 in G1 phase is involved in Dpb2 interaction with Pol2. This is supported by the finding that *DPB2* mutant in Cdc28 CDK sites demonstrate as a synthetic defect with a Pol2 mutant in the C-terminus (*pol2-11*) which interacts with Dpb2 (Kesti et al. 2004). Furthermore, the excessive dephosphorylation of Dpb2 in a Cdc14 mutant strain combined with mutations in CDK sites of Dpb2 severely impairs cell viability (Bloom and Cross 2007). Therefore, although there was no severe phenotypic effect of mutations in Dpb2 CDK sites, it cannot be excluded that the cell cycle-dependent phosphorylation of Dpb2 is involved in other aspects of DNA replication and the cell cycle progression.

Given that Dpb2 is involved in DNA replication initiation, is phosphorylated in a cell cycle-dependent manner and that the *dpb2*-*103* mutant demonstrates a different pattern of G1/S transition genes transcription, it might be speculated that Dpb2 is also involved in the regulatory processes during replication initiation. How does it happen? Firstly, the Dpb2 protein, which is phosphorylated by Cdc28, may directly influence G1/S genes expression by yet unknown mechanisms. Secondly, Dpb2 may be involved in the communication between the assembling replisomes and MBF regulators. This would define a mechanism coordinating the pre-IC complexes assembly and G1/S gene expression prior to the transition to the S phase. Finally, the observed anomalies in transcriptional repression of G1/S transition genes may constitute a more indirect cellular response to DNA replication (initiation) perturbation that occurs in the *DPB2* mutant (this hypothesis is not favored given that derepression of MBF-dependent genes in *nrm1*Δ cells suppresses, at least partially, some of *dpb2*-*103* phenotypes)—(Fig. 5 in Dmowski et al. 2017). Strikingly, the premature exit of *dpb2*-*103* cells from the G1 phase precedes a prolonged S phase observed in the analyses of DNA content (FACS) and G1/S-specific transcripts after the release from G1 block—(Fig. 6 in Dmowski et al. 2017). The premature entry into S phase (when not enough origins have been licensed) and the prolonged S phase have been observed as a result of the overexpression of Cln2 or deletion of the Sic1 inhibitor of Clb5-6 (Lengronne and Schwob 2002). This effect can be alleviated by the deletion of *CLB5* and *CLB6* (Lengronne and Schwob 2002). Because the MBF factor regulates various genes involved in DNA replication, their premature repression may also, at least partially, contribute to the observed S phase perturbations, the mutagenic effect of mutations in *DPB2*, and alleviation of *dpb2*-*103* phenotypes by *nrm1*Δ. However, the elucidation of how the mutations in *DPB2* affect the regulation of MBF-dependent genes and, as a consequence, genomic stability, needs further investigations.
